# Prevalence of hepatitis among young men who have sex with men and transgender women in Brazil

**DOI:** 10.11606/s1518-8787.2024058005555

**Published:** 2024-10-11

**Authors:** Carina Carvalho dos Santos, Beo Oliveira Leite, Fernanda Washington de Mendonça Lima, Laio Magno, Alexandre Grangeiro, Mateus Westin, Daniel Lima de Moura, Inês Dourado

**Affiliations:** I Universidade Federal da Bahia. Faculdade de Farmácia. Departamento de Análises Clínicas e Toxicológicas. Salvador, BA, Brasil Universidade Federal da Bahia Faculdade de Farmácia Departamento de Análises Clínicas e Toxicológicas Salvador BA Brasil; II Universidade Federal da Bahia. Instituto de Saúde Coletiva. Salvador, BA, Brasil Universidade Federal da Bahia Instituto de Saúde Coletiva Salvador BA Brasil; III Universidade do Estado da Bahia. Departamento de Ciências da Vida. Salvador, BA, Brasil Universidade do Estado da Bahia Departamento de Ciências da Vida Salvador BA Brasil; IV Universidade de São Paulo. Faculdade de Medicina. São Paulo, SP, Brasil Universidade de São Paulo Faculdade de Medicina São Paulo SP Brasil; V Universidade Federal de Minas Gerais. Faculdade de Medicina. Belo Horizonte, MG, Brasil Universidade Federal de Minas Gerais Faculdade de Medicina Belo Horizonte MG Brasil

**Keywords:** Men who have Sex with Men, Hepatitis A, Hepatitis B, Hepatitis C, Prevalence, Adolescents

## Abstract

**OBJECTIVE:**

Viral hepatitis and sexually transmitted infections disproportionally affect men who have sex with men (MSM) and transgender women (TGW). However, only a few studies have evaluated the prevalence of hepatitis in these populations, especially in youths and adolescents. This study aimed to estimate the prevalence of biomarkers for hepatitis A, B, and C among young and adolescent MSM and TGW in three Brazilian municipalities.

**METHODS:**

Baseline data were collected from a combination of HIV prevention cohort of young and adolescent MSM (AMSM) and TGW (ATGW) aged 15-19 years in three Brazilian municipalities. A social behavioral questionnaire was applied, and immunoassays were performed to detect antibodies against hepatitis A (anti- HAV IgG and IgM), hepatitis B (anti-HBc and anti-HBs), and hepatitis C virus (anti-HCV); testing for the active hepatitis B marker, HBsAg, was also performed. The prevalence of reactive tests and 95% confidence interval (CI) for proportions were measured.

**RESULTS:**

The prevalence of naturally or artificially acquired immunity for hepatitis A totaled 17.7% (95%CI: 15.4-20.4), whereas that of acute infection, 0.4% (95%CI: 0.2-1.2). For hepatitis B and C, prevalence rates totaled 2.8% (95%CI: 1.8-4.4) and 0.2% (95%CI: 0.1-1.1), respectively. About 25.7% (95%CI: 22.4-29.4) of participants were non-reactive for anti-HBc and reactive for anti-HBs, the latter being a vaccine marker for hepatitis B.

**CONCLUSIONS:**

The investigation of viral hepatitis biomarkers among vulnerable populations enables the early identification of infections, the provision of timely treatment, and an opportunity to point out the need to expand vaccination coverage.

## INTRODUCTION

Populations whose living status poses an increased risk and vulnerability for Human Immunodeficiency Virus (HIV) infection, such as men who have sex with men (MSM) and transgender women (TGW) are also more vulnerable and at greater risk of being infected by some types of the hepatitis virus, such as the hepatitis A (HAV), B (HBV), and C viruses (HCV)^1^^), (^^2^^), (^^3^^), (^^4^^), (^^5^. An HAV outbreak with low endemicity occurred among MSM and TGW in some European and American countries in 2016-2017^6^^), (^^7^. An increase in HAV seroprevalence in the general population also occurred in Brazil^8^. It is noteworthy that immunization for HAV became available in the Brazilian public health system for children in 2014, which was later expanded to MSM in 2018 in the state of São Paulo. More recently, it has been expanded to serve the entire national territory^9^^), (^^10^^), (^^11^. The practice of oral-anal, digital-anal, and genital-oral sex involving a partner with HAV may contribute to concentrating cases in this population^12^^), (^^13^. A study conducted in France with people taking pre-exposure prophylaxis (PrEP) found six patients with acute hepatitis A in 38 individuals who were yet to be vaccinated for the infection, considering it a high incidence rate^14^.

HBV infection also occurs to a considerable extent in MSM and TGW populations^15^^), (^^16^^), (^^17^. Approximately 20% of new cases of HBV infection in adults occur among gay and bisexual men in the United States (US)^15^. Moreover, higher rates of hepatitis B have been reported in specific MSM subgroups, such as gay and bisexual homeless young adults in the US (52.4%)^16^. A systematic review with meta-analysis conducted in 2022 indicated a global hepatitis B prevalence of 11% in TGW^8^.

HCV frequency has also increased among MSM and TGW. Transmission can occur by sexual practices that increase the risk of blood-blood contact (e.g., anal sex and fisting)^18^. A previous systematic review with a meta-analysis showed a higher prevalence of HCV among MSM living with HIV infections than HIV-negative MSM^19^. Another study showed a global HCV seroprevalence of 9% in TGW^3^. A study conducted from October 2005 to October 2006 with MSM living in Campinas, São Paulo, Brazil, showed that 11.4% and 1% of individuals were infected with HBV and HCV, respectively^20^.

Few studies have evaluated the prevalence of viral hepatitis in adolescent and young MSM and TGW in Brazil, especially regarding hepatitis A. Thus, this study describes markers of acute, past, or chronic viral hepatitis A, B, and C and of artificially acquired immunity by vaccination for hepatitis B in adolescent MSM (AMSM) and transgender women (ATGW) aged 15-19 years who participated in an HIV pre-exposure prophylaxis demonstration study (PrEP1519).

## METHODS

### Study Design, Data Collection, and Ethical Aspects

This cross-sectional descriptive study used data from participants who were enrolled in the baseline cohort of the PrEP1519 project, the first study to show the effectiveness of PrEP in AMSM and ATGW aged 15-19 years in Latin America. It was conducted in three large Brazilian capitals: Salvador, São Paulo, and Belo Horizonte. That study was approved by the ethical review board of the World Health Organization (Fiotec-PrEP Adolescent study) and the local committees in each municipality: the Federal University of Bahia in Salvador (Protocol ID: 3,224,384), the University of São Paulo in São Paulo (Protocol ID: 3,082,360), and the Federal University of Minas Gerais in Belo Horizonte (Protocol ID: 2,027,889)^21^.

Volunteers were invited to participate in this study, sign informed consent forms, and answer a sociodemographic and sociobehavioral questionnaire containing questions related to their lifestyle, sexual practices, experiences of violence or discrimination, and the strategies they used to prevent sexually transmitted infections (STIs). A special judicial authorization was obtained to waive the requirement for parental consent for adolescents aged <18 years. In São Paulo, this authorization enabled total waiver of consent; in Salvador, the waiver was partial and only applied to adolescents at risk of violence due to the disclosure of their sexuality to their parents or guardians and in cases of family breakdown (which were analyzed by professionals); in Belo Horizonte, all participants aged <18 years were required to obtain consent from their legal guardians.

This study consisted of a non-probabilistic sampling of 1,129 adolescents from the baseline cohort (362 from the PrEP1519-Salvador site, 248 from the PrEP1519-Belo Horizonte site, and 519 from the PrEP1519-São Paulo site) who were enrolled in the study from February 2019 to June 2021. The following were chosen as inclusion criteria: being AMSM or ATGW; aged 15-19 years at the time of admission; having sex with cis men or trans women; and residing, working, or studying in one of the three aforementioned Brazilian capitals, whereas the following criteria were chosen for exclusion: renal impairment (i.e., defined as a glomerular filtration rate below 60 ml/min/1.75 m^2^ according to the Cockcroft-Galt formula for adolescents aged over 17 years and the Schwartz formula for those aged under 17 years); a history of bone fractures without any obvious cause; symptoms of acute retroviral syndrome in the previous 30 days; and engagement in high-risk sexual activity in the previous 72 hours (i.e., requiring immediate treatment with PEP).

The convenience sampling of this study is highlighted as participants are linked to a PrEP cohort study and generally more exposed to sexual intercourse. The number of testing centers varied depending on the municipality of the study and the number of participants who agreed to be tested for the different immunological markers for viral hepatitis, which made it difficult to obtain accurate data.

### Evaluation of Immunological Markers for Viral Hepatitis

Blood samples were collected from the cubital vein by a trained professional using a vacuum tube with gel separator and clot activator (BD Vacutainer® SST™). Immunochromatography assays, Bioclin and ABON kits, were performed to detect the active hepatitis B marker (HBsAg) and the antibodies against HCV (total anti-HCV), respectively

After collection and rapid testing, the samples were centrifuged at 3,000 rpm for 10 min at room temperature to separate the serum, which was stored at -20ºC until testing. The antibodies against HAV (anti-HAV IgG and anti-HAV IgM), HBV (total anti-hepatitis B core antibody (HBc) and anti-HBs), and HCV (total anti-HCV) were evaluated using the enzyme-linked immunosorbent assay (Dia.pro Diagnostic Bioprobes Srl).

Although 1,129 adolescents were involved in this study and responded to its questionnaire, the number of participants who agreed to be tested for the different immunological markers for viral hepatitis varied: 903 participants were tested for the anti-HAV IgG marker; 918, for anti-HAV IgM; 936, for HBsAg; 643, for total anti-HBc; 903, for anti-HBs; and 836, for total anti-HCV.

### Study Variables

The variables based on participants’ answers to the sociobehavioral questionnaire were categorized as follows: gender identity (AMSM and ATGW), age (15-17 and 18-19 years), education level (primary, secondary, and higher education), race/skin color (White, Mixed race, Black, and others), employed (no or yes), living arrangement (with parents and/or family members or not), participation in NGOs/social movements (no or yes), usual sources of healthcare (public, private, unofficial, or none), health insurance status (none or yes), condom use during first sexual intercourse (no or yes), condom use during insertive/receptive anal sex in the last six months (irregular or regular use), STI episode in the past six months (no or yes), PEP use in the last 12 months (no or yes), use of alcohol/drug before or during sex in the last six months (no or yes), alcohol consumption in the last three months (no or yes), use of any illicit drugs in the last three months (no or yes), use of injectable drugs in the last three months (no or yes), needle sharing (no or yes), experience of discrimination and/or violence related to gender identity or sexual orientation (no or yes), experience of physical assault (no or yes), experience of forced sex (no or yes).

The viral hepatitis profile was determined as follows:

- Hepatitis A
  - Acute infection: positive reaction for anti-HAV IgM.
  - Naturally or artificially acquired immunity: positive reaction for anti-HAV IgG.
- Hepatitis B
  - Naturally acquired immunity (past infection): positive reaction for total anti-HBc and anti-HBs and negative reaction for HBsAg.
  - Artificially acquired immunity by vaccination: positive reaction for anti-HBs only and negative reaction for the other HBV markers.
  - Active infection (present): positive reaction for HBsAg.
- Hepatitis C
  - Present or past infection: positive reaction for total anti-HCV.

### Data Analysis

The absolute and relative frequencies and respective 95% confidence intervals (95%CIs) were estimated for each variable. Missing or atypical values were disregarded. Artificially acquired immunity by vaccination for Hepatitis B (non-reactive for anti-HBc and reactive for anti-HBs) was investigated as an outcome in an exploratory analysis for associated factors. Bivariate logistic regression was performed considering significant confounders with a *p*-value level ≤0.05 and a 95%CI to account for them in a multiple logistic regression analysis. Stata, v. 15.0, (Stata Corporation, College Station, USA) was used for data tabulation.

## RESULTS

The baseline cohort of this study included 1,129 adolescents, of whom 1,031 (91.3%) identified as AMSM and 78 (8.7%), as ATGW. Most were aged 18 or 19 years (79.4%), had completed secondary education (68.5%), and identified as Black (39.4%). At the time of this study, 44.5% of respondents reported being employed or working in informal jobs; 76.7% lived with parents or relatives, and 11.4% participated in NGOs and/or social movements. Regarding the main source of healthcare, 67.6% reported using public sources, and only 26.2% had purchased a health insurance (Table 1).

**Table 1:** Description of the sample of AMSM and ATGW who participated in the PrEP 15-19 study, February 2019 to June 2021.

Characteristic	Total (N=1,129)		
	n	%	95%CI
Gender identity			
AMSM	1,031	91.3	89.5-92.8
ATGW	78	8.7	7.2-10.5
Age			
15-17 years	223	20.6	18.4-23.1
18-19 years	896	79.4	76.9-81.6
Education			
Primary education	69	6.6	5.2-8.2
Secondary education	719	68.5	65.7-71.3
Higher education	261	24.9	22.4-27.6
Race/skin color			
White	314	27.8	25.3-30.5
Mixed-race	334	29.6	27.0-32.3
Black	445	39.4	36.6-42.3
Other	36	3.2	2.3-4.4
Currently employed			
No	594	55.5	52.5-58.4
Yes	477	44.5	41.6-47.5
With whom they currently live			
With parents and/or relatives	886	76.7	74.1-79.1
Not with parents and/or relatives	263	23.3	20.9-25.9
Participation in NGOs/social movements			
No	947	88.6	86.5-90.4
Yes	122	11.4	9.6-13.5
Usual source of healthcare			
Public sources	721	67.6	64.7-70.3
Private sources	175	16.4	14.3-18.7
Unofficial sources	152	14.2	12.3-16.5
None	19	1.8	1.1-2.8
Health insurance			
No	778	73.8	71.1-76.4
Yes	276	26.2	23.6-28.9
Condom use during first intercourse			
No	543	52.8	49.8-55.9
Yes	485	47.2	44.1-50.2
Condom use during insertive/receptive anal sex, last six months			
Yes	223	19.7	17.5-22.2
No	906	80.3	77.8-82.5
STI episode, last six months			
No	810	79.0	76.4-81.4
Yes	215	21.0	18.6-23.6
PEP use, last 12 months			
No	928	90.5	88.6-92.2
Yes	97	9.5	7.8-11.4
Alcohol/drugs use before or during sexual intercourse			
No	702	68.5	65.6-71.3
Yes	323	31.5	28.7-34.4
Alcohol consumption, last three months			
Never	126	11.8	10.0-13.9
At least once a month or less	236	88.2	86.1-90.0
Use of any illicit drug, last three months			
Never	545	51.1	48.1-54.1
At least once a month or less	521	48.9	45.9-51.9
Use of needles and syringes to inject drugs, last three months			
No	538	99.3	98.0-99.7
Yes	4	0.7	0.3-2.0
Needle sharing			
No	6	85.7	25.7-99.5
Yes	1	14.3	1.0-74.3
Discrimination and violence related to affective-sexual life			
No	679	66.2	63.3-69.1
Yes	346	33.8	30.9-36.7
Physical assault, last six months			
No	1,006	94.2	92.6-95.5
Yes	62	5.8	4.5-7.4
Forced sex, ever			
No	756	71.6	68.8-74.2
Yes	300	28.4	25.8-31.2

Approximately 47.2% of adolescents reported using condoms during their first sexual intercourse, whereas most (80.3%) reported using no condoms during insertive and/or receptive anal sex in the last six months; 21.0% reported having some type of STIs in the last six months, and 9.5% used PEP in the last 12 months. Regarding alcohol and drug use, 88.2% had consumed alcohol at least once in the last three months, 48.9% had used an illicit drug, and 31.5% reported consuming alcohol or using other drugs before or during sexual intercourse. Only 0.7% of the participants used needles and syringes to inject drugs, whereas one reported sharing a needle or syringe with others. Worryingly, 33.8% of participants frequently experience discrimination and violence; 5.8%, physical assault related to their gender or sexual orientation in the last six months, and 28.4%, forced sex (Table 1).

The estimated prevalence of naturally or artificially acquired immunity for hepatitis A totaled 17.7% (95%CI: 15.4-20.4%); whereas that for acute infection, 0.4% (95%CI: 0.2-1.2%). About 2.8% (95%CI: 1.8-4.4%) had a hepatitis B virus infection (total anti-HBc reactive), and 0.2% (95%CI: 0.1-1.0%) were positive for total anti-HCV. Analysis of the hepatitis B markers showed that 25.7% (95%CI: 22.4-29.4%) of participants were non-reactive for anti-HBc and reactive for anti-HBs (vaccine marker), and none had an active infection at the time of sample collection (Table 2). The exploratory analysis (hepatitis B vaccine marker and associated factors) in this study found no significant results for the bivariate analysis. Thus, the subsequent multiple logistic regression analysis included no variables (Table 3).

**Table 2:** Profile of viral hepatitis markers in PrEP 15-19 participants, February 2019 to June 2021.

Marker	Result	n (%)	95%CI
**HAV**			
Anti-HAV IgG	Non-reactive	743 (82.3)	79.6-84.6
	Reactive	160 (17.7)	15.4-20.4
Anti-HAV IgM	Non-reactive	914 (99.6)	98.8-99.8
	Reactive	4 (0.4)	0.2-1.2
HBV			
HBsAg	Non-reactive	936 (100.0)	
	Reactive	0 (0.0)	
Total anti-HBc	Non-reactive	625 (97.2)	95.6-98.2
	Reactive	18 (2.8)	1.8-4.4
Anti-HBs	Non-reactive	609 (67.4)	64.3-70.4
	Reactive	294 (32.6)	29.6-35.7
Anti-HBs (non-reactive for anti-HBc)	Non-reactive	453 (74.3)	70.6-77.6
	Reactive	157 (25.7)	22.4-29.4
HCV			
Total anti-HCV	Non-reactive	834 (99.8)	99.0-99.9
	Reactive	2 (0.2)	0.1-1.0

Note: HAV: hepatitis A virus; HBV: hepatitis B virus; HCV: hepatitis C virus; CI: confidence interval.

**Table 3:** Bivariate analysis of the factors associated with hepatitis B vaccination according to age group, PrEP1519, February 2019 to June 2021.

Characteristic	15-17 years				18-19 years			
	Vaccinated against hepatitis B				Vaccinated against hepatitis B			
	%	P-value	OR	95%CI	%	P-value	OR	95%CI
Gender identity		0.552^a^				0.182^a^		
MSM	26.09		-	-	23.73		-	-
TGW	33.33		1.42	0.45-4.48	32.61		1.56	0.81-2.99
Socioeconomic level		0.091^a^				0.701^a^		
Low	39.53		-	-	25.00		-	-
Medium	16.67		0.31	0.10-.095	27.18		1.12	0.64-1.96
High	26.00		0.54	0.22-1.29	22.94		0.89	0.56-1.43
Education		1.000^b^				0.869^b^		
Primary education	25.93		-	-	18.75		-	-
Secondary education	28.09		1.12	0.42-2.96	23.53		1.33	0.37-4.80
Higher education	33.33		1.43	0.11-18.30	25.21		1.46	0.39-5.47
Race/skin color		0.852^a^				0.802^a^		
White	28.57		-	-	24.45		-	-
Other	26.61		0.91	0.32-2.56	24.29		0.94	0.58-1.53
Currently employed		0.165^a^				0.517^a^		
No	32.89		-	-	25.55		-	-
Yes	21.28		0.55	0.34-1.29	23.00		0.87	0.53-1.32
With whom they currently live		0.893^a^				0.055^a^		
With parents and/or relatives	26.67		-	-	22.77		-	-
Not with parents and/or relatives	28.00		1.07	0.40-2.83	32.26		1.61	0.99-2.64
Participation in NGOs/social movements		0.211^a^				0.594^a^		
No	26.21		-	-	24.15		-	-
Yes	40.00		1.88	0.69-5.08	27.66		1.20	0.61-2.36
Usual source of healthcare		0.487^a^				0.441^a^		
Official sources	28.81		-	-	23.77		-	-
Unofficial sources/none	21.05		0.63	0.19-2.04	27.85		1.23	0.72-2.13
Health insurance		0.373^b^				0.421^a^		
No	27.88		-	-	25.63		-	-
Yes	35.71		1.44	0.44-0.65	22.05		0.82	0.50-1.33
Condom use during first intercourse		0.954^a^				0.710^a^		
No	26.47		-	-	24.07		-	-
Yes	26.00		0.98	0.43-2.24	25.55		1.08	0.71-1.65
Condom use during insertive/receptive anal sex, last six months		0.971^a^				0.951^a^		
No	26.67		-	-	24.32		-	-
Yes	27.00		1.02	0.40-2.56	24.61		1.02	0.62-1.66
STI episode, last six months		0.599^a^				0.710^a^		
No	27.27		-	-	22.91		-	-
Yes	33.33		1.33	0.45-3.91	24.73		1.11	0.65-1.88
Use of alcohol/drugs before or during sexual intercourse		0.383^a^				0.703^a^		
No	25.64		-	-	23.83		-	-
Yes	33.33		1.45	0.63-3.35	22.22		0.91	0.57-1.45
Gender or sexual orientation-based discrimination		0.294^a^				0.233^a^		
No	25.00		-	-	25.00		-	-
Yes	34.15		1.56	0.68-3.56	20.00		0.75	0.47-1.20
Use of any illicit drug, last three months		0.934^a^				0.914^a^		
No	28.99		-	-	24.24		-	-
Yes	28.30		0.97	0.44-2.14	24.66		1.02	0.68-1.55
Alcohol consumption, last three months		0.517^a^				0.666^a^		
Never	31.25		-	-	22.00		-	-
At least once	28.57		0.88	0.28-2.75	24.77		1.17	0.58-2.36
Physical assault		0.418^a^				0.372^a^		
No	29.20		-	-	24.68		-	-
Yes	20.00		0.61	0.12-3.01	16.67		0.61	0.20-1.82
Forced sex, ever		0.413^a^				0.140^a^		
No	31.03		-	-	26.35		-	-
Yes	23.53		0.68	0.27-1.70	19.85		0.69	0.42-1.13

MSM: men who have sex with men; TGW: transgender women; NGO: non-governmental organization; STI: sexually transmitted infection. ^a^Pearson’s chi-squared test. ^b^Fisher’s exact test.

## DISCUSSION

According to the WHO, MSM and TGW configure key populations for HIV prevention and are disproportionally affected by viral hepatitis and STIs. Thus, the literature has increasingly recognized the importance of addressing all three infectious disease areas in an integrated, community-led, and person-centered manner^22^. Accordingly, this study assessed the prevalence of viral hepatitis among AMSM and ATGW in Brazil.

The incidence rate of hepatitis A in Brazil has shown a downward trend, from 3.9 to 0.2 cases per 100,000 from 2011 to 2021, a decrease of 95.6%^23^. Unfortunately, few studies have evaluated the prevalence of hepatitis A among MSM and TGW in Brazil, especially in youths. A previous study conducted in these populations (aged 18-70 years) in Campo Grande, in the state of Mato Grosso do Sul, Brazil, from November 2011 to September 2013 reported that 40.0% of MSM and 79.2% of TGW (<20 years) had undergone previous HAV exposure and that one participant was positive for anti-HAV IgM^24^. Another study conducted from 2018 to 2019 among TGW (with a median age of 25 years) living in three municipalities in Goiás, in Midwest Brazil, showed that 64.03% of participants aged ≤21 years had been exposed to HAV^25^. Thus, the data on previous exposure to HAV or vaccination (17.7%) in our study is smaller than in other studies conducted in Brazil; whereas its data on acute infection (0.4%) agrees with the literature.

However, it is important to note that the number of participants aged 15-19 years in the aforementioned studies^24^^), (^^25^ is smaller than in this study. Although less likely, the different health policies (e.g., hepatitis A vaccination policies for MSM) from where the studies were conducted may lead to differences in prevalence rates. The Brazilian National Immunization Plan has included Hepatitis A vaccination for children aged 15-24 months since 2014; expanding its coverage to children aged ≤5 years in 2017^9^^), (^^10^. From August 2014 to December 2018, vaccination coverage against hepatitis A in children ranged from 60.13% to 97.07% in Brazil. Although the years following 2015 witnessed a drop in coverage, the incidence of hepatitis A in all age groups decreased, which may be related to herd immunity^26^. Vaccination against hepatitis A has been recommended for populations whose sexual practices involve oral-anal contact, especially among those who seek the Centers for Testing and Counseling and the Services that offer PEP, PrEP, or other treatments aimed at controlling STIs in São Paulo, Brazil^11^.

Moreover, a previous study with the general population of three Brazilian regions showed that hepatitis A prevalence varied according to region and age (individuals aged 5-9 and 10-19 years). Overall, prevalence rates ranged from 30.8% to 58.3% in individuals aged 5-19 years^27^. According to the WHO classification, the levels of endemicity were divided based on seroprevalence rates: intermediate level ≥50% by age 15 years and <90% by age 10 years and low level ≥50% by age 30 years and <50% by age 15 years^28^.

This study found that 25.7% of participants were non-reactive for anti-HBc and reactive for anti-HBs (vaccination marker), 2.8% were reactive for total anti-HBc (marker of previous exposure to the virus), and none were actively infected (HBsAg reactive). These data corroborate other studies with MSM and TGW populations. A study conducted in Campinas, Brazil, among MSM, from 2005 to 2006, reported that around 32% displayed a serological profile compatible with a vaccine response (anti-HBs alone)^20^. A study conducted with TGW in three cities in Goiás, in Midwest Brazil, from 2018 to 2019 reported a prevalence of 41.5% for the vaccine marker for hepatitis B and that 12.3% of the TGW were exposed to HBV^25^, a high prevalence when compared with the data in this study. Another study with MSM, conducted from March to November 2014 in Goiânia, Goiás, showed that 40.3% of participants were vaccinated for hepatitis B^29^. The percentage of the population in this study that was reactive for anti-HBs is only below the vaccination coverage of the general population in Brazil: 66.03% from 2019 to 2021^30^. It is important to mention that vaccine-induced anti-HBs levels may decline over time^31^. Thus, the data in this study may be underestimated. This researched failed to verify the vaccination records of its participants.

Unprotected sex, drug use, and sex work may also configure a risk factor for acquiring HCV, which is currently considered an STI, especially related to anal sex^13^. Injectable drug use is also a significant risk factor for HCV infection, of which this study reported a low frequency (0.7%). The prevalence of hepatitis C (0.2%) agrees with other studies conducted among MSM and TGW in Brazil^20^^), (^^25^ and other countries^14^. Diagnosing hepatitis C remains a challenge^32^. Thus, monitoring the markers of infection in at-risk populations, such as MSM and TGW, is of the utmost importance, including the use of techniques to detect viral RNA and monitor reinfections in those with positive serology.

Other STIs have been assessed and showed relatively high prevalence rates among the AMSM and ATGW population from the PrEP1519 study^33^^), (^^34^. This study has some limitations. Anti-HBs data as a marker for vaccination may be underestimated (as previously discussed). The data on the prevalence of viral hepatitis markers, combined with a small sample size, may have made it difficult to perform a robust analysis of the associated factors. We also highlight the convenience sampling of this study as participants are linked to a PrEP cohort study and are generally more exposed to sexual intercourse. Regarding the statistical analysis, the impact of sample size remained the same as that of the PrEP study^21^ in this study evaluating viral hepatitis. Its average post-hoc power remained below 50% to estimate associations. Thus, we are unable to rule out the possibility of a type II error. Moreover, the number of testing centers varied depending on the municipality of the study and the number of participants who agreed to be tested for the immunological markers for viral hepatitis, which made it difficult to obtain the accurate data.

## Data Availability

The datasets used and/or analyzed in this study are available from the corresponding author upon reasonable request.

## References

[1] Latash J, Dorsinville M, Del Rosso P, Antwi M, Reddy V, Waechter H (2017). Notes from the Field: Increase in Reported Hepatitis A Infections Among Men Who Have Sex with Men - New York City, January-August 2017. MMWR Morb Mortal Wkly Rep.

[2] Wirtz AL, Yeh PT, Flath NL, Beyrer C, Dolan K (2018). HIV and Viral Hepatitis Among Imprisoned Key Populations. Epidemiol Rev.

[3] Moradi G, Soheili M, Rashti R, Dehghanbanadaki H, Nouri E, Zakaryaei F (2022). The prevalence of hepatitis C and hepatitis B in lesbian, gay, bisexual and transgender populations: a systematic review and meta-analysis. Eur J Med Res.

[4] Van Gerwen OT, Jani A, Long DM, Austin EL, Musgrove K, Muzny CA (2020). Prevalence of Sexually Transmitted Infections and Human Immunodeficiency Virus in Transgender Persons: A Systematic Review. Transgend Health.

[5] Charre C, Ramiere C, Roque-Afonso AM, Chidiac C, Zoulim F, Godinot M (2017). Hepatitis A outbreak in HIV-infected MSM and in PrEP-using MSM despite a high level of immunity, Lyon, France, January to June 2017. Euro Surveill.

[6] World Health Organization (2017). Hepatitis A outbreaks mostly affecting men who have sex with men - European Region and the Americas [Internet].

[7] Ndumbi P, Freidl GS, Williams CJ, Mardh O, Varela C, Avellon A (2018). Hepatitis A outbreak disproportionately affecting men who have sex with men (MSM) in the European Union and European Economic Area, June 2016 to May 2017. Euro Surveill.

[8] São Paulo, Coordenadoria de Vigilância em Saúde (2019). Boletim Epidemiológico n° 9 - Hepatite A [Internet].

[9] Brasil, Ministério da Saúde, Secretaria de Vigilância em Saúde, Departamento de Vigilância Epidemiológica, Coordenação Geral do Programa Nacional de Imunizações (2014). Informe técnico da introdução da vacina adsorvida hepatite A (inativada).

[10] Brasil, Ministério da Saúde, Secretaria de Vigilância em Saúde, Departamento de Vigilância das Doenças Transmissíveis (2016). Nota informativa nº 311, de 2016/CGPNI/DEVIT/SVS/MS [Internet].

[11] Brasil, Ministério da Saúde (2018). Nota Informativa nº 10/2018-COVIG/CCVP/.DIAHV/SVS/MS: ampliação da indicação do uso da vacina de Hepatite A para pessoas que tenham prática sexual com contato oral-anal (com priorização de gays e homens que fazem sexo com homens (HSH).

[12] Bialek SR, Barry V, Bell BP, Valleroy LA, Behel S, Mackellar DA (2011). Seroprevalence and correlates of hepatitis A among HIV-negative American men who have sex with men. Sex Health.

[13] Bouvet E (2005). Sexual practices and transmission of HAV and HCV. Euro Surveill.

[14] Migueres M, Ducours M, Dimeglio C, Trimoulet P, Abravanel F, Delobel P (2020). No evidence of sexual transmission of HEV among individuals using HIV pre-exposure prophylaxis. Journal Viral Hepat.

[15] Centers for Disease Control and Prevention (2019). Viral Hepatitis - Information for Gay and Bisexual Men [Internet].

[16] Nyamathi A, Salem B, Reback CJ, Shoptaw S, Branson CM, Idemundia FE (2013). Correlates of hepatitis B virus and HIV knowledge among gay and bisexual homeless young adults in Hollywood. Am J Mens Health.

[17] Bastos FI, Bastos LS, Coutinho C, Toledo L, Mota JC, Velasco-de-Castro CA (2018). HIV, HCV, HBV, and syphilis among transgender women from Brazil: assessing different methods to adjust infection rates of a hard-to-reach, sparse population. Medicine (Baltimore).

[18] Vaux S, Chevaliez S, Saboni L, Sauvage C, Sommen C, Barin F (2019). Prevalence of hepatitis C infection, screening and associated factors among men who have sex with men attending gay venues: a cross-sectional survey (PREVAGAY), France, 2015. BMC Infect Dis.

[19] Jin F, Dore GJ, Matthews G, Luhmann N, Macdonald V, Bajis S (2021). Prevalence and incidence of hepatitis C virus infection in men who have sex with men: a systematic review and meta-analysis. Lancet Gastroenterol Hepatol.

[20] Soares CC, Georg I, Lampe E, Lewis L, Morgado MG, Nicol AF (2014). HIV-1, HBV, HCV, HTLV, HPV-16/18, and Treponema pallidum infections in a sample of Brazilian men who have sex with men. PloS One.

[21] Dourado I, Magno L, Greco DB, Zucchi EM, Ferraz D, Westin MR (2023). Interdisciplinarity in HIV prevention research: the experience of the PrEP1519 study protocol among adolescent MSM and TGW in Brazil. Cad Saude Publica.

[22] World Health Organization (2022). Consolidated guidelines on HIV, viral hepatitis and STI prevention, diagnosis, treatment and care for key populations.

[23] Brasil, Ministério da Saúde, Secretaria de Vigilância em Saúde (2022). Boletim Epidemiológico de Hepatites Virais 2022.

[24] Castro LS, Rezende GR, Fernandes FRP, Bandeira LM, Cesar GA, Lago BV (2021). HAV infection in Brazilian men who have sex with men: the importance of surveillance to avoid outbreaks. PloS One.

[25] Ferri LP, Junqueira PS, Almeida MMS, Oliveira MG, Oliveira BR, Silva BVD (2022). Viral Hepatitis A, B and C in a Group of Transgender Women in Central Brazil. Trop Med Infect Dis.

[26] Brito WI, Souto FJD (2020). Universal hepatitis A vaccination in Brazil: analysis of vaccination coverage and incidence five years after program implementation. Rev Bras Epidemiol.

[27] Pereira LMMB, Stein AT, Figueiredo GM, Coral GP, Montarroyos UR, Cardoso MRA (2021). Prevalence of hepatitis A in the capitals of the States of North, Southeast and South regions of Brazil: decrease in prevalence and some consequences. Rev Inst Med Trop Sao Paulo.

[28] Giovanetti M, Fonseca V, Navegantes W, Abreu A, Stabeli R, Vazquez CC (2023). Strengthening genomic surveillance capacity for epidemic-prone pathogens by training personnel from public health laboratories in Brazil and Paraguay. Wkly Epidemiol Rec.

[29] Oliveira MP, Matos MAD, Silva AMC, Lopes CLR, Teles SA, Matos MA (2016). Prevalence, Risk Behaviors, and Virological Characteristics of Hepatitis B Virus Infection in a Group of Men Who Have Sex with Men in Brazil: Results from a Respondent-Driven Sampling Survey. PloS One.

[30] Brasil, Ministério da Saúde, Secretaria de Vigilância em Saúde, Departamento de Vigilância Epidemiológica (2005). Sistema de Informação do Programa Nacional de Imunizações.

[31] Ren W, Ren J, Wu Z, Shen L, Shan H, Dai X (2020). Long-term persistence of anti-HBs after hepatitis B vaccination among adults: 8-year results. Hum Vaccin Immunother.

[32] World Health Organization (2017). Guidelines on Hepatitis B and C Testing.

[33] Magno L, Medeiros DS, Soares F, Grangeiro A, Caires P, Fonseca T (2023). Factors associated to HIV prevalence among adolescent men who have sex with men in Salvador, Bahia State, Brazil: baseline data from the PrEP1519 cohort. Cad Saude Publica.

[34] Westin MR, Martinez YF, Silva AP, Greco M, Marques LM, Campos GB (2023). Prevalence of syphilis and sexual behavior and practices among adolescents MSM and TrTGW in a Brazilian multi-center cohort for daily use of PrEP. Cad Saude Publica.

