# Erratum

**DOI:** 10.11606/s1518-8787.2025059005555err

**Published:** 2025-06-27

In the article “Prevalence of hepatitis among young men who have sex with men and transgender women in Brazil”, DOI http://doi.org/10.11606/s1518-8787.2020054005555, published on the Revista de Saúde Pública. 2024;58: Suppl 1:4s, RSP corrects:

**How to cite (page 1):**

Where it reads:

http://doi.org/10.11606/s1518-8787.2020054005555

It should read:

http://doi.org/10.11606/s1518-8787.2024058005555

**Footnote (pages 1 to 12):**

Where it reads:

http://doi.org/10.11606/s1518-8787.2020054005555

It should read:

http://doi.org/10.11606/s1518-8787.2024058005555

